# Ocular Neurofibromatosis

**DOI:** 10.7759/cureus.17765

**Published:** 2021-09-06

**Authors:** Saba Alkhairy, Mahad M Baig

**Affiliations:** 1 Ophthalmology, Dow International Medical College, Dow University of Health Sciences, Karachi, PAK

**Keywords:** neurofibromatosis type 1 (nf-1), plexiform neurofibromas, upper blepharoplasty, eyelid mass, eye surgery, orbital, eye ptosis, eyelid neurofibroma

## Abstract

A 14-year-old boy presented with a right orbital lid mass, which had slowly grown over the last 4.5 years, as well as some impaired visual acuity in the affected (right) eye. We assessed the patient by taking a detailed history and physical examination. A Snellen chart was used to assess visual acuity, which revealed decreased acuity in the right eye as compared to the left eye. Pupillary reactions, including relative afferent pupillary reflexes, were unremarkable; anterior and posterior chamber assessment was normal including that of the optic disc and macula. Additionally, the intraocular pressure was within acceptable limits. The mass was excised surgically as it had caused significant disfigurement and posed risk to the patient in terms of the possibility for the lesion to increase in severity. It was an approach utilizing a blepharoplasty incision, horizontal wedge resection, and a frontalis sling done under general anesthesia. A biopsy of the mass identified it as a plexiform lesion of the orbit such as that attributed to neurofibromatosis type 1.

## Introduction

Neurocutaneous pathologies typically arise during embryonic development as congenital disorders of neuroectodermal or mesodermal origin. They present as abnormalities of the skin, nervous system, and eye [1]. Neurofibromatosis (NF) is a genetic disorder observed with relative prevalence among neurocutaneous syndromes [2]. Two forms of this genetic disorder may be delineated: neurofibromatosis type 1 (NF1) and neurofibromatosis type 2 (NF2). They may be classified according to their chromosomal pathogenesis: NF1 arises from a mutation on chromosome 17, causing dysfunction of the neurofibromin protein; NF2 arises from a mutation on chromosome 22, resulting in dysfunction of the merlin protein [3]. Neurofibromatosis type 1 is also known as von Recklinghausen's disease [4], representing the overwhelming majority of NF cases [5] and the focus of this report. NF1 additionally carries with it a vast spectrum of clinical presentations, which mainly consist of neurofibromas, macules, cafe-au-lait spots, and optic gliomas [4], whereas a hallmark of NF2 is the manifestation of bilateral vestibular schwannomas. Neither NF1 nor NF2 demonstrates a predilection to any particular sex or ethnic group, but NF1 has a higher incidence overall. NF2, in contrast to NF1, is associated with such a high tumor burden that it may result in reduced life expectancy of the afflicted [3].

Due to the incurable nature of neurofibromatosis type 1 and 2, symptomatic treatment is currently the only effective management of this disease. In the realm of ophthalmology, the most pertinent of physical manifestations include the following ocular signs: plexiform and conjunctival neurofibromas, corneal stromal nerve hypertrophy (lignes grise), iris hamartoma (Lisch nodules), early-onset cataracts, glaucoma, patchy choroidal appearance due to choroidal nodules, corkscrewing of retinal vasculature/retinal hamartomas, and optic nerve gliomas causing proptosis/strabismus [6]. Hence, ophthalmic intervention is a much-desired therapy for many patients for a variety of reasons: plexiform and conjunctival neurofibromas can both cause eye-lid mechanical ptosis resulting in impaired vision and facial disfigurement, the former of which is observed in this report, early-onset cataracts may result in vision loss, and congenital glaucoma may progress to cause buphthalmos - ultimately leading to loss of the eye itself.

Before procedures can be undertaken, a diagnosis must be confirmed through the appropriate evaluations such as radiographic imaging (CT/MRI), infrared fundus autofluorescence (IR-FAF), optical tomography, scintigraphy, electroencephalogram (EEG), and genetic analysis. The results may thereafter warrant therapy of varying invasiveness such as pharmacotherapy, phototherapy, chemotherapy, electrodesiccation, and surgical therapies [7]. Recent advances in laser technologies bring better opportunities for patients with this condition to experience a higher quality of life and a better prognosis than previously possible.

## Case presentation

**Figure 1:**
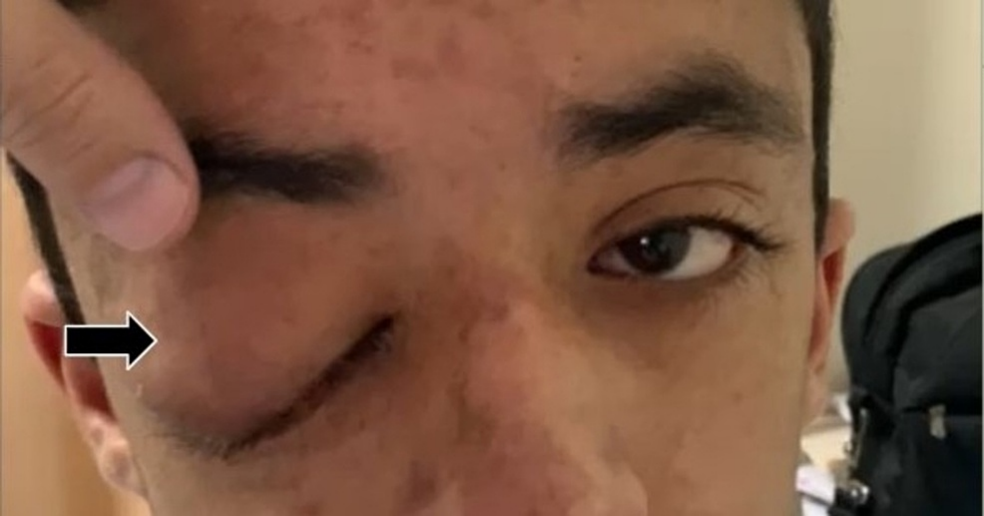
Right orbital lid mechanical ptosis

A 14-year-old boy presented to the eye out-patient department (OPD) of Dow University of Health Sciences (DUHS) in Karachi, Pakistan, with right lid mechanical ptosis (Figure 1), which was painless for the past 4.5 years. He denied any history of exacerbation of symptoms, visual impairments, associated cephalgia, trauma, emesis, nausea, or fever. Systemic inquiry yielded no significant findings such as a loss of appetite, weight loss, lethargy, bone pain, and skin lesions, with the exception of scattered cafe-au-lait spots across his abdomen of various sizes and the aforementioned solitary nodule, which conform to the diagnosis of NF1.

**Figure 2:**
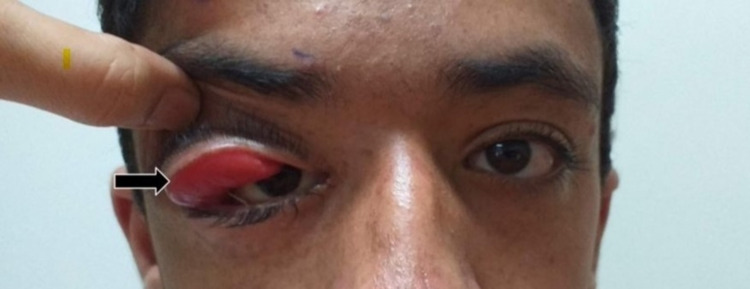
Upturned right orbital lid

On examination, the right eye (oculus dexter [OD]) mechanical ptosis was prominent. A high degree of suspicion was attributed to the mass bulking the same eyelid, as observed upon lifting it to display the bulging mucosal surface underneath (Figure 2). Although no thrill was noted in the OD, the large mass was rubbery, soft, and immobile on palpation measuring 3 x 5 cm, and was markedly visible within the blepharon. Furthermore, the texture could be described as a “bag of worms.” Transillumination was negative. On palpation, the left eye (oculus sinister [OS]) and blepharon provided no significant findings with no mass and no thrill noted. An anterior segment examination of the left eye was unremarkable with no sign of chemosis, edematous cornea, dilation, or tortuous conjunctival vessels. On the other hand, OD revealed Lisch nodules on slit-lamp microscopy during the anterior segment exam with no other abnormalities detected. Pupillary reflexes were unremarkable with no relative afferent pupillary defect in both eyes (oculus uterque [OU]). Posterior segment examination was clear in both eyes with no disc/macular edema or other abnormalities. Extraocular movements were unaffected OU, and intraocular pressure (IOP) was unremarkable in both eyes. Visual acuity was noted at 6/12 OD and 6/6 OS. Both preauricular, as well as submandibular lymph nodes, were palpated; no enlargement was detected. Subsequent CT scan imaging confirmed the presence of a peripherally enhanced mass involving the right orbital blepharon as a result of significant neurocutaneous expansion causing downwards displacement of the orbit.

**Figure 3:**
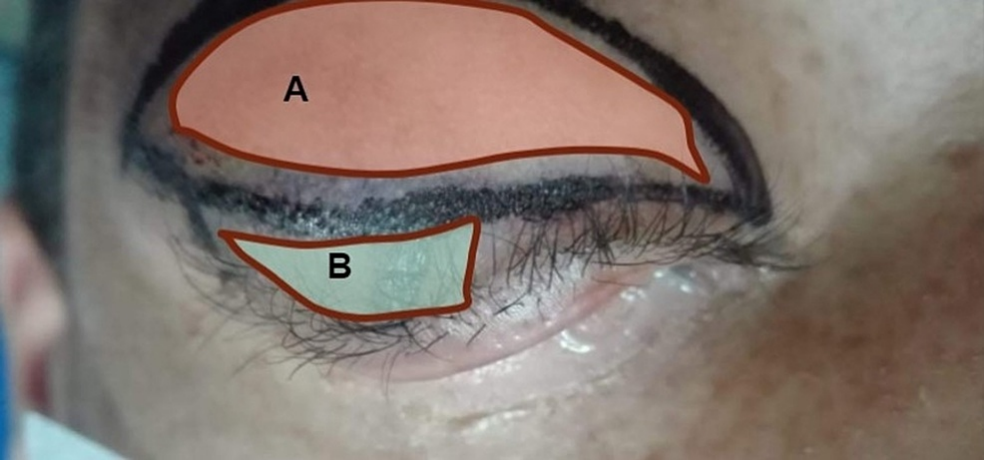
Pre-operative photograph of the blepharoplasty incision and horizontal wedge resection area The area shaded in red (A) denotes the area of the blepharoplasty incision and the area shaded in green (B) denotes the area of the horizontal wedge resection.

**Figure 4:**
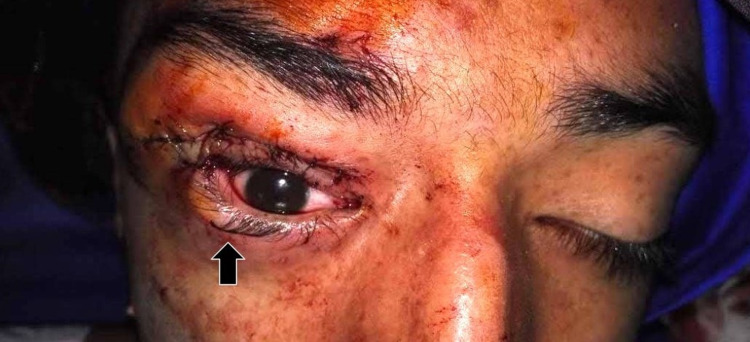
An immediate post-operative photograph of the patient

As this image warranted surgery, proper protocols were followed to evaluate and prepare the patient after confirmation that admittance criteria were met. First, an incision was performed directly inferior to the eyebrow following the length of the infraorbital ridge to gain access to the superior orbit and the frontoethmoid region. Next, a blepharoplasty incision (Figure 3A) was made to debulk the mass. Next, a horizontal wedge resection (Figure 3B) was completed to shorten the horizontal laxity at the lid margin on the lateral side, and a frontalis sling to elevate the lid was implemented. The excised mass was sent for a biopsy. A postoperative picture is provided in Figure 4.

The patient was discharged per hospital procedures and given sufficient discharge instructions on broad-spectrum antibiotics taken orally, steroids, and antibacterial ointment to be applied topically for 10 days. He fully recovered from surgery with no complications after a period of one week as confirmed on the patient’s return to the OPD for follow-up. Histological analysis of the biopsy revealed a neurofibroma with its structure favoring the presence of a plexiform lesion consistent with the preliminary diagnosis of NF1.

## Discussion

The characteristic neurofibromas in NF1 are outgrowths that can be classified as any of the three types: cutaneous, subcutaneous, or plexiform [8]. Cutaneous type growths are only a few millimeters in length and mostly non-tender. Subcutaneous neurofibromas, on the other hand, are tender to manipulation and measure approximately 3 to 4 centimeters. Notably, both cutaneous and subcutaneous neurofibromas are non-specific for NF1. In such cases, the diagnosis must be carefully decided based on the presence of other signs and symptoms. This is in contrast to plexiform neurofibromas, which have the greatest tendency to transform into malignant peripheral nerve sheath tumors (MPNST), including malignant schwannoma, neurofibrosarcoma, and neurogenic sarcoma; they may also have the potential to cause significant hemifacial hypertrophy [8]. It is most likely that a plexiform lesion was to blame for this patient’s right eyelid mechanical ptosis, and while surgical intervention is not the most desired form of therapy for most patients, it is a crucial point of preventative care to avoid the formation of MPNSTs when dealing with a case of NF1.

Fortunately, recent advances in laser therapy have the potential to provide desirable outcomes for patients afflicted with NF1 without invasive procedures. An example of such recent innovations includes that of a free-hand carbon dioxide (CO2) laser, which operates in continuous mode (10-20 W, incorporating fast-paced hand movements to either side). It may also be operated in scanner settings (125 mm handpiece, 7-14 W, 3 mm spot) for the ablation of smaller neurofibromas [9]. The larger lesions are first scored at their peripheries with the laser beam in continuous mode. The incision is then expanded while holding the neurofibroma using forceps. Finally, the whole dumbbell-shaped lesion is excised using the laser. This type of therapy is thereby able to avoid most complications associated with alternatively invasive treatments. A study evaluated the efficacy of CO2 laser ablation on NF1 patients concluding that this treatment has great potential to reduce the social impact of this disease as patients opted for this procedure mostly for aesthetic reasons [10].

In some cases, plexiform neurofibromas may be inoperable, leaving patients with few options for treatment. In fact, until very recently, there were no approved therapies for inoperable plexiform lesions. The first of such therapies included the development and subsequent approval of Koselugo (selumetinib) by the United States Food and Drug Administration (USFDA) in 2020. This drug is a mitogen-activated kinase kinase (MEK) inhibitor used for the purpose of inhibiting the overactivation of RAS proteins, which occurs as a result of dysfunctional neurofibromin in NF1 patients [11]. Such therapy may reduce tumor size in patients of NF1 who are otherwise helpless.

## Conclusions

In conclusion, this 14-year-old boy was afflicted with ocular NF1 and was subsequently evaluated for surgical treatment in order to resolve his prominent mechanical right eye ptosis. This approach allowed for recovery of the patient’s comfort by way of restoring his ability to open and close the eye with relative ease. There is also potential for improvement in overall visual acuity. Although an invasive surgical intervention such as that which was performed in this case was necessary per the urgent circumstances of this patient, recent medical advances pave the way for less invasive treatment options to become viable for patients like this one.
